# Random forest-driven mortality prediction in critical IBD care: a dual-database model integrating comorbidity patterns and real-time physiometrics

**DOI:** 10.3389/fmed.2025.1624899

**Published:** 2025-08-08

**Authors:** Zhenze Zhang, Caiqing Zhao, Yijun Zhou, Ling Yao, Peng Liu, Ziling Fang, Nian Fang

**Affiliations:** ^1^Clinical Graduate School, Jiangxi Medical College, Nanchang University, Nanchang, China; ^2^The 1st Affiliated Hospital, Nanchang University, Nanchang, China; ^3^The 3rd Affiliated Hospital (The First Hospital of Nanchang), Nanchang University, Nanchang, China; ^4^Clinical Graduate School, Jiangxi University of Traditional Chinese Medicine, Nanchang, China

**Keywords:** machine learning, mortality prediction, critical care, nomogram, inflammatory bowel disease

## Abstract

**Background:**

Inflammatory bowel disease (IBD) poses significant mortality risks for critically ill patients requiring intensive care unit (ICU) admission, driven by complications such as malnutrition, thromboembolism, and multi-organ dysfunction. Current prognostic tools for mortality prediction in this population remain limited. Machine learning (ML) offers advantages in handling complex clinical data but has not been systematically applied to this high-risk cohort. This multicenter study aimed to develop and validate ML-based models for mortality risk stratification in critically ill IBD patients using large-scale ICU databases.

**Methods:**

Data from 551 IBD patients in the MIMIC-IV database (2008–2019) were analyzed, with external validation using the eICU dataset. Nine ML algorithms (XGBoost, logistic regression, LightGBM, random forest, decision tree, elastic net, MLP, KNN, RSVM) were trained to predict 1-year mortality. Predictors included demographics, comorbidities, laboratory parameters, vital signs, and disease severity scores. Missing data (<30%) were imputed using random forest. The cohort was split into training (75%) and internal testing (25%) sets, with hyperparameter optimization via 5-fold cross-validation. Model performance was evaluated using AUC, sensitivity, specificity, and calibration curves. The SHAP framework was integrated with predictive analytics to systematically evaluate key determinants of mortality risk through quantitative feature importance analysis. A nomogram was constructed based on key predictors identified through logistic regression.

**Results:**

The random forest model achieved superior discrimination in internal validation (AUC > 0.8). Nine predictors were identified: malignancy history, Charlson Comorbidity Index (CCI), Red Cell Distribution Width (Rdw), Glasgow Coma Scale (GCS), Sequential Organ Failure Assessment (Sofa), age, heart rate, weight and gender. The nomogram demonstrated robust external validation performance in the eICU cohort (AUC > 0.8).

**Conclusion:**

We developed and validated a machine learning-based nomogram to predict mortality in critically ill IBD patients, integrating interpretable predictors from multicenter ICU data.

## Introduction

Inflammatory bowel disease (IBD), a condition characterized by chronic relapsing intestinal inflammation, presents a significant burden to patients (1). The common gastrointestinal manifestations encompass hematochezia, persistent diarrhea, and abdominal discomfort (2). Beyond these, sufferers may also contend with extra-intestinal symptoms, which extend to cutaneous, ocular, and joint inflammations (3). In spite of extensive investigations and remarkable therapeutic headways, IBD endures as a refractory chronic condition, exerting a profound and enduring impact on the quality of life and overall well-being of affected individuals (4, 5).

Population-based cohort studies have established that IBD-related mortality primarily stems from nutritional deficiencies, hypovolemic shock, refractory anemia, infectious complications, malignancy development, and postoperative sequelae (6, 7). Malnutrition represents a particularly prevalent concern in severe IBD cases, with critical illness exacerbating preexisting nutritional deficits. Furthermore, these patients demonstrate heightened thromboembolic risks secondary to surgical interventions and prolonged immobilization (8). Notably, severe ulcerative colitis has been associated with multiple organ dysfunction syndrome (MODS), frequently necessitating intensive care unit (ICU) admission (9). Current evidence indicates ICU mortality rates ranging from 15 to 19% among critically ill IBD patients (10). Previous investigations have identified key prognostic determinants including advanced age, mechanical ventilation requirement, acute renal failure development, and prior immunomodulatory therapy exposure (11–13).

Machine learning (ML), an emerging artificial intelligence technology, has demonstrated increasing utility in medical data analytics (14, 15). Compared with conventional statistical approaches, ML algorithms exhibit superior predictive performance in specific clinical scenarios, particularly in critical care settings (16, 17). Nevertheless, no existing studies have developed ML-based models for mortality prediction in critically ill IBD populations. This study therefore aims to develop and validate innovative ML algorithms to enhance mortality risk stratification in this vulnerable patient cohort.

## Methods

### Data sources and study design

This retrospective cohort study utilized data from the Medical Information Mart for Intensive Care IV version 2.0 (MIMIC-IV v2.0) database (18), jointly maintained by the Beth Israel Deaconess Medical Center and the Massachusetts Institute of Technology. The database contains de-identified medical records of >70,000 intensive care unit (ICU) admissions spanning 2008 to 2019, encompassing comprehensive clinical data including laboratory parameters, therapeutic interventions, pharmacological treatments, diagnostic codes (International Classification of Diseases, 9th/10th revisions), and physiological monitoring records (19). Data access authorization was obtained through completion of the required training courses (Certification ID: 68554343). The study protocol adhered to the TRIPOD (Transparent Reporting of a Multivariable Prediction Model for Individual Prognosis or Diagnosis) guidelines for predictive model development and validation (20).

**Figure 1:**
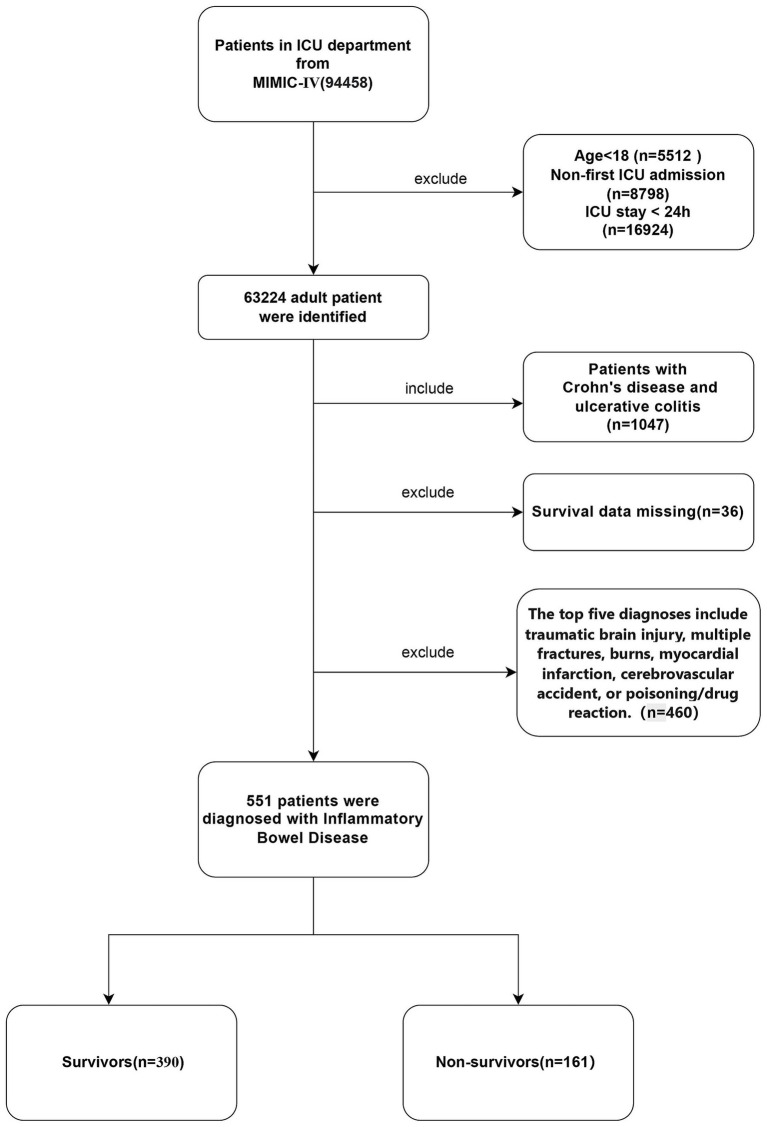
Flow diagram of the selection process of patients in MIMIC IV.

**Figure 2:**
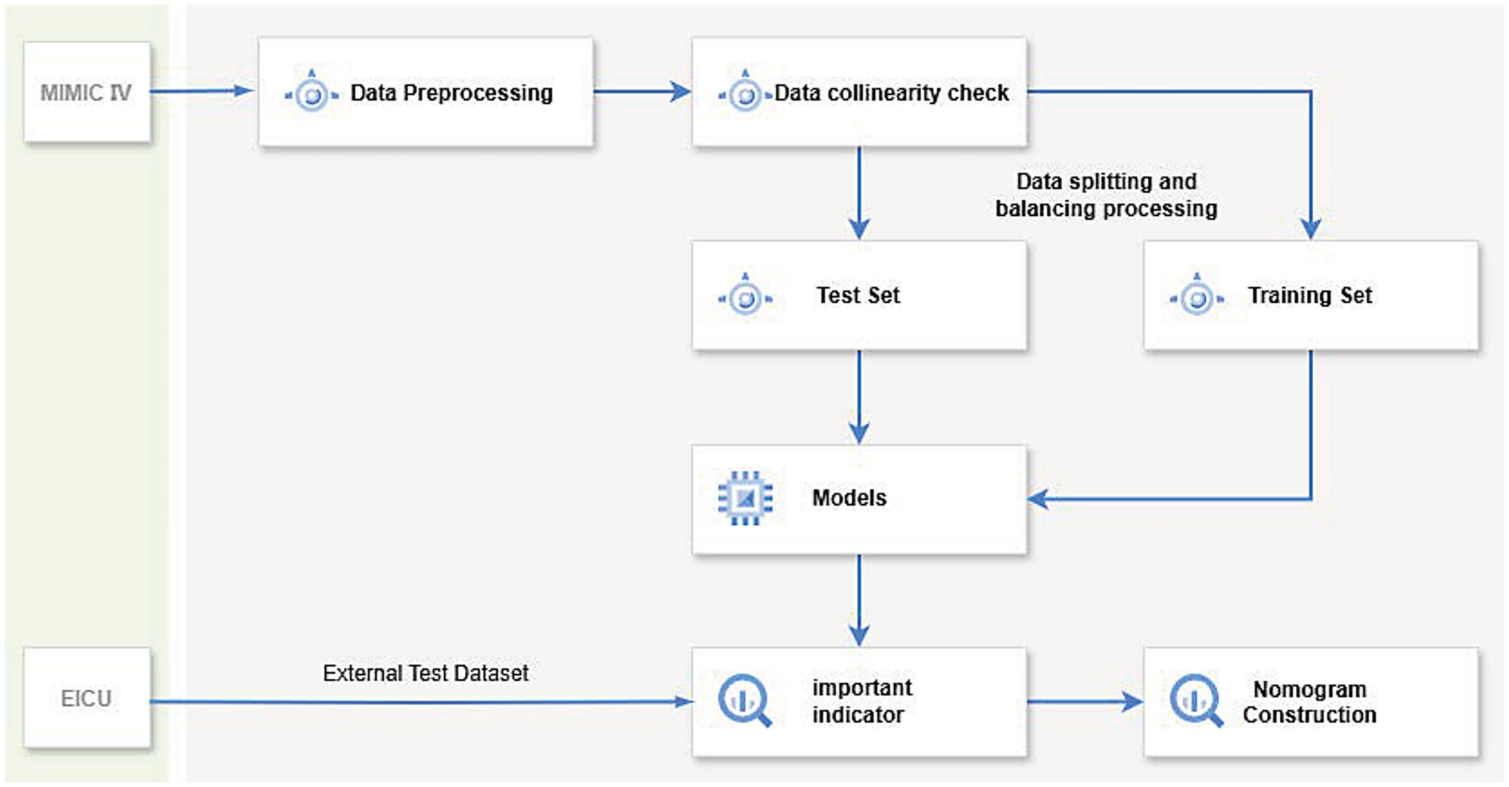
The diagram illustrates the workflow of a machine learning model: first, data preprocessing and collinearity checks are performed on the dataset, which is then split into a training set and a test set. After balancing the data, various machine learning methods are applied to train the model, evaluate key indicators, and validate using the eICU dataset. Finally, a nomogram is constructed to predict the 1-year mortality rate of patients with severe inflammatory bowel disease.

Initially, 1,047 adult patients with inflammatory bowel disease (IBD) (aged ≥18 years) who were admitted to the intensive care unit (ICU) for the first time during the study period were included. To ensure the integrity and accuracy of the data, the following exclusion criteria were established: (1) patients with an ICU stay of less than 24 h; (2) patients with the first five diagnoses including traumatic brain injury, multiple fractures, burns, myocardial infarction, cerebrovascular accident, or poisoning/medication reaction; (3) patients with incomplete follow-up records or laboratory data missing by more than 30%. After applying the aforementioned exclusion criteria, a total of 551 eligible patients were included in the analysis. The patients included in the final analysis were divided into two groups according to their mortality status during hospitalization: the survival group (n = 390) and the non-survival group (*n* = 161). Figures 1, 2 illustrate the study design framework and the patient selection process, respectively.

### Data collection

In this study, six categories of potential variables were extracted using Structured Query Language (SQL) commands via PostgreSQL (version 13.7.2) and Navicat Premium (version 16) software. These categories were as follows: (1) Demographic data, including age at admission, sex, height, weight, history of alcohol abuse, and smoking status; (2) Comorbidities, such as hypertension, diabetes, liver diseases, acute kidney injury, malignant tumors, clostridioides difficile infection, intestinal dysfunction, melena, rheumatic diseases, and sepsis; (3) Laboratory indicators, covering white blood cell count, absolute values and percentages of various blood cells, red blood cell distribution width, platelet count, and biochemical parameters; (4) Vital signs, including heart rate, respiratory rate, blood pressure, and body temperature; (5) Treatment measures, such as the use of immunosuppressants, glucocorticoids, antihypertensive medications, invasive ventilation, cardiopulmonary resuscitation, renal replacement therapy, and colonoscopy; (6) Disease severity scores at admission, including the Glasgow Coma Scale (GCS), Sequential Organ Failure Assessment (SOFA), and Charlson Comorbidity Index (CCI). To ensure data integrity, missing values were managed systematically. Variables with more than 30% missing data were excluded. For variables with less than 30% missing values, multiple imputation was performed using the Random Forest (RF) algorithm within the “mice” package of R software. The imputation process utilized other non-missing variables as the training basis, thereby reducing potential bias and ensuring the quality of the dataset for subsequent analysis.

### Clinical outcomes

The primary outcome is all-cause mortality within 1 year after admission. These survival data were extracted from the MIMIC-IV and eICU databases.

### Statistical analyses

We compared the above characteristics between survivor and non-survivor groups and between training and validation cohorts in the MIMIC database. Continuous variables are expressed as median and interquartile range (IQR) and were compared using t-tests. Count data were presented as numbers and percentages and compared using chi-square tests.

Given the demonstrated superiority of machine learning (ML) algorithms in handling high-dimensional datasets compared to conventional regression approaches (21), we implemented nine distinct ML models: Extreme Gradient Boosting (XGBoost), logistic regression, Light Gradient Boosting Machine (LightGBM), Random Forest (RF), Decision Tree (DT), Elastic Net, Multilayer Perceptron (MLP), k-Nearest Neighbors (KNN), and Relevance Vector Support Machine (RSVM). A total of 75% of the samples were randomly selected for model training, while the remaining 25% were used for testing. Moreover, no statistically significant differences were observed in any baseline characteristics between the two groups (*p* > 0.05) (Supplementary Table 1). Throughout the training process, we focused on parameter tuning to prevent overfitting and determined the optimal hyperparameters of the machine learning models using five-fold cross-validation. Subsequently, we further trained these machine learning algorithms based on the R language to predict the risk of adverse outcomes in IBD. We evaluated the predictive ability of each machine learning classifier using the test set, calculating the area under the receiver operating characteristic curve (AUC) as well as the corresponding sensitivity, specificity, and overall accuracy. When comparing the performance of machine learning algorithms, an AUC value closer to 1 indicates better classification model performance. Ultimately, we selected the risk factors identified by the optimal algorithm and constructed a nomogram. The discrimination and calibration of the nomogram were assessed using concordance statistics and calibration curves, respectively. Additionally, we validated the identified risk factors using data from IBD patients in the eICU database and constructed ROC curves. All tests were two-sided, with *p*-values less than 0.05 considered statistically significant. All statistical analyses were performed using R version 4.0.4 and SPSS version 16.0 (SPSS Inc., Chicago, IL, USA).

## Results

### Baseline characteristics

The study enrolled a total of 551 patients, with an all-cause mortality rate of 29.2% at 365 days. Table 1 shows significant differences between the two groups at baseline. A total of 551 patients were included in this study, which were divided into the survival group (390 cases) and the non-survival group (161 cases) based on the outcomes. The analysis revealed that the non-survival group had a significantly higher mean age and a higher proportion of females compared with the survival group. There were no significant differences between the two groups in terms of smoking status and alcohol consumption. Regarding comorbidities, the incidence of severe liver disease and malignant tumors was significantly higher in the non-survivor group than in the survivor group. In terms of therapeutic interventions, the proportion of patients receiving renal replacement therapy was significantly higher in the non-survival group, whereas the proportion of patients receiving immunosuppressive therapy was significantly lower. No significant differences were observed between the two groups in the use of invasive ventilation, cardiopulmonary resuscitation, glucocorticoids, antihypertensive drugs, or colonoscopy. Comparison of physiological and laboratory parameters showed that the non-survival group had a significantly lower body weight and a significantly higher heart rate. Hematological indicators revealed that the non-survival group had significantly lower hemoglobin levels, a significantly higher red cell distribution width, and a significantly lower platelet count. Moreover, the non-survival group had significantly higher levels of blood urea nitrogen, international normalized ratio, alkaline phosphatase, and absolute monocyte count.

**Table 1:** Baseline characteristics of the survivors and non-survivors groups.

	Survivors (*N* = 390)	Non-survivors (*N* = 161)	p.overall
Demographic			
Age (years)	60.1 (14.4)	67.6 (13.9)	<0.001
Gender (%):			0.005
Female	165 (42.3%)	90 (55.9%)	
Male	225 (57.7%)	71 (44.1%)	
Smoker (%):			0.864
No	360 (92.3%)	150 (93.2%)	
Yes	30 (7.69%)	11 (6.83%)	
Alcohol abuse (%):			0.327
No	386 (99.0%)	161 (100%)	
Yes	4 (1.03%)	0 (0.00%)	
Comorbidities			
Hypertension (%):			1
No	268 (68.7%)	110 (68.3%)	
Yes	122 (31.3%)	51 (31.7%)	
Diabetes (%):			0.05
No	306 (78.5%)	113 (70.2%)	
Yes	84 (21.5%)	48 (29.8%)	
Mild liver disease (%):			0.072
No	308 (79.0%)	115 (71.4%)	
Yes	82 (21.0%)	46 (28.6%)	
Severe liver disease (%):			0.024
No	345 (88.5%)	130 (80.7%)	
Yes	45 (11.5%)	31 (19.3%)	
Renal disease (%):			0.23
No	315 (80.8%)	122 (75.8%)	
Yes	75 (19.2%)	39 (24.2%)	
Malignant cancer (%):			<0.001
No	367 (94.1%)	127 (78.9%)	
Yes	23 (5.90%)	34 (21.1%)	
Rheumatic disease (%):			0.645
No	369 (94.6%)	150 (93.2%)	
Yes	21 (5.38%)	11 (6.83%)	
*Clostridium difficile* infection (%):			0.284
No	355 (91.0%)	141 (87.6%)	
Yes	35 (8.97%)	20 (12.4%)	
Intestinal dysfunction (%):			0.365
No	387 (99.2%)	158 (98.1%)	
Yes	3 (0.77%)	3 (1.86%)	
Black stool (%):			0.085
No	376 (96.4%)	149 (92.5%)	
Yes	14 (3.59%)	12 (7.45%)	
Sepsis (%):			0.074
No	167 (42.8%)	55 (34.2%)	
Yes	223 (57.2%)	106 (65.8%)	
Aki (%):			0.051
No	129 (33.1%)	39 (24.2%)	
Yes	261 (66.9%)	122 (75.8%)	
Treatments			
InvasiveVent (%):			0.078
No	251 (64.4%)	90 (55.9%)	
Yes	139 (35.6%)	71 (44.1%)	
Cpr (%):			0.206
No	389 (99.7%)	159 (98.8%)	
Yes	1 (0.26%)	2 (1.24%)	
Rrt (%):			0.012
No	357 (91.5%)	135 (83.9%)	
Yes	33 (8.46%)	26 (16.1%)	
Immunosuppressant (%):			0.022
No	344 (88.2%)	153 (95.0%)	
Yes	46 (11.8%)	8 (4.97%)	
Corticosteroids (%):			0.974
No	230 (59.0%)	94 (58.4%)	
Yes	160 (41.0%)	67 (41.6%)	
Antihypertensive drugs (%):			0.364
No	151 (38.7%)	55 (34.2%)	
Yes	239 (61.3%)	106 (65.8%)	
Colonoscopy (%):			0.859
No	376 (96.4%)	154 (95.7%)	
Yes	14 (3.59%)	7 (4.35%)	
Vital signs and laboratory results			
Temperature (°C)	36.7 (0.87)	36.7 (1.00)	0.541
Weight (kg)	81.1 (20.2)	73.5 (20.5)	<0.001
Heart rate (times/min)	91.2 (21.4)	98.7 (21.0)	<0.001
Resp rate (times/min)	19.8 (6.17)	20.7 (6.02)	0.09
Sbp (mmHg)	116 (21.3)	118 (23.0)	0.342
Dbp (mmHg)	66.3 (15.1)	68.8 (18.3)	0.12
Wbc (×109/L)	13.0 (13.2)	13.2 (11.1)	0.818
Absolute eosinophils count (K/uL)	0.08 (0.14)	0.10 (0.19)	0.295
Absolute basophils count (K/uL)	0.02 (0.03)	0.03 (0.08)	0.714
Absolute lymphocytes count (K/uL)	1.13 (1.24)	1.04 (0.94)	0.32
Absolute neutrophils count (K/uL)	10.3 (6.70)	12.0 (11.0)	0.072
Absolute monocytes count (K/uL)	0.58 (0.45)	0.94 (1.67)	0.007
Eosinophils (%)	0.87 (1.73)	0.94 (1.49)	0.647
Monocytes (%)	5.36 (4.12)	6.67 (7.13)	0.03
Neutrophils (%)	77.6 (15.6)	76.9 (17.7)	0.663
Basophils (%)	0.23 (0.29)	0.25 (0.56)	0.648
Lymphocytes (%)	11.7 (10.7)	11.8 (13.7)	0.996
Rdw (%)	15.6 (2.78)	16.8 (2.79)	<0.001
Platelet (×10^9^/L)	235 (145)	201 (139)	0.01
Creatinine (mg/dL)	1.59 (1.91)	1.68 (1.90)	0.59
Bun (mg/dL)	26.8 (25.0)	34.0 (29.8)	0.008
Anion gap (mEq/L)	14.4 (4.83)	15.2 (5.16)	0.099
Calcium total (mg/dL)	8.20 (0.88)	8.24 (1.31)	0.738
Pt(sec)	17.4 (11.2)	19.7 (13.3)	0.054
INR	1.59 (0.92)	1.91 (1.56)	0.017
Alt (IU/L)	72.7 (172)	86.9 (407)	0.669
Ast (IU/L)	114 (306)	175 (931)	0.413
Alp (IU/L)	117 (115)	150 (138)	0.007
Bilirubin total(mg/dL)	2.25 (5.25)	3.39 (6.78)	0.058
Chloride(mEq/L)	103 (7.22)	102 (8.12)	0.592
Glucose(mEq/L)	140 (77.1)	150 (88.9)	0.244
Potassium(mEq/L)	4.07 (0.71)	4.07 (0.89)	0.979
Sodium(mEq/L)	137 (5.36)	136 (6.36)	0.791
Hemoglobin(g/dL)	10.5 (2.36)	9.81 (2.13)	0.002
Magnesium(g/dL)	1.94 (0.51)	1.98 (0.42)	0.378
Phosphate(g/dL)	3.69 (1.63)	3.88 (1.94)	0.266
Disease severity			
Sofa	5.18 (3.74)	7.73 (5.02)	<0.001
Gcs	13.5 (2.80)	11.8 (4.24)	<0.001
Charlson	3.94 (2.70)	5.90 (2.55)	<0.001

Aki, Acute Kidney Injury; InvasiveVent, Invasive Ventilation; Cpr, Cardiopulmonary Resuscitation; Rrt, Renal Replacement Therapy; Sbp, Systolic Blood Pressure; Dbp, Diastolic Blood Pressure; Resp rate, Respiratory rate; Wbc, White Blood Cell Count; Platelet, Rdw, Red Cell Distribution Width; Bun, Blood Urea Nitrogen; Pt, Partial Thromboplastin Time; INR, International Normalized Ratio; Alt, Alanine Aminotransferase; Ast, Aspartate Aminotransferase; Alp, Alkaline Phosphatase; Sofa, Sequential Organ Failure Assessment; Gcs, Glasgow Coma Scale; Charlson, Charlson Comorbidity Index.

### Machine learning algorithms and comparison statistical prediction model

**Figure 3:**
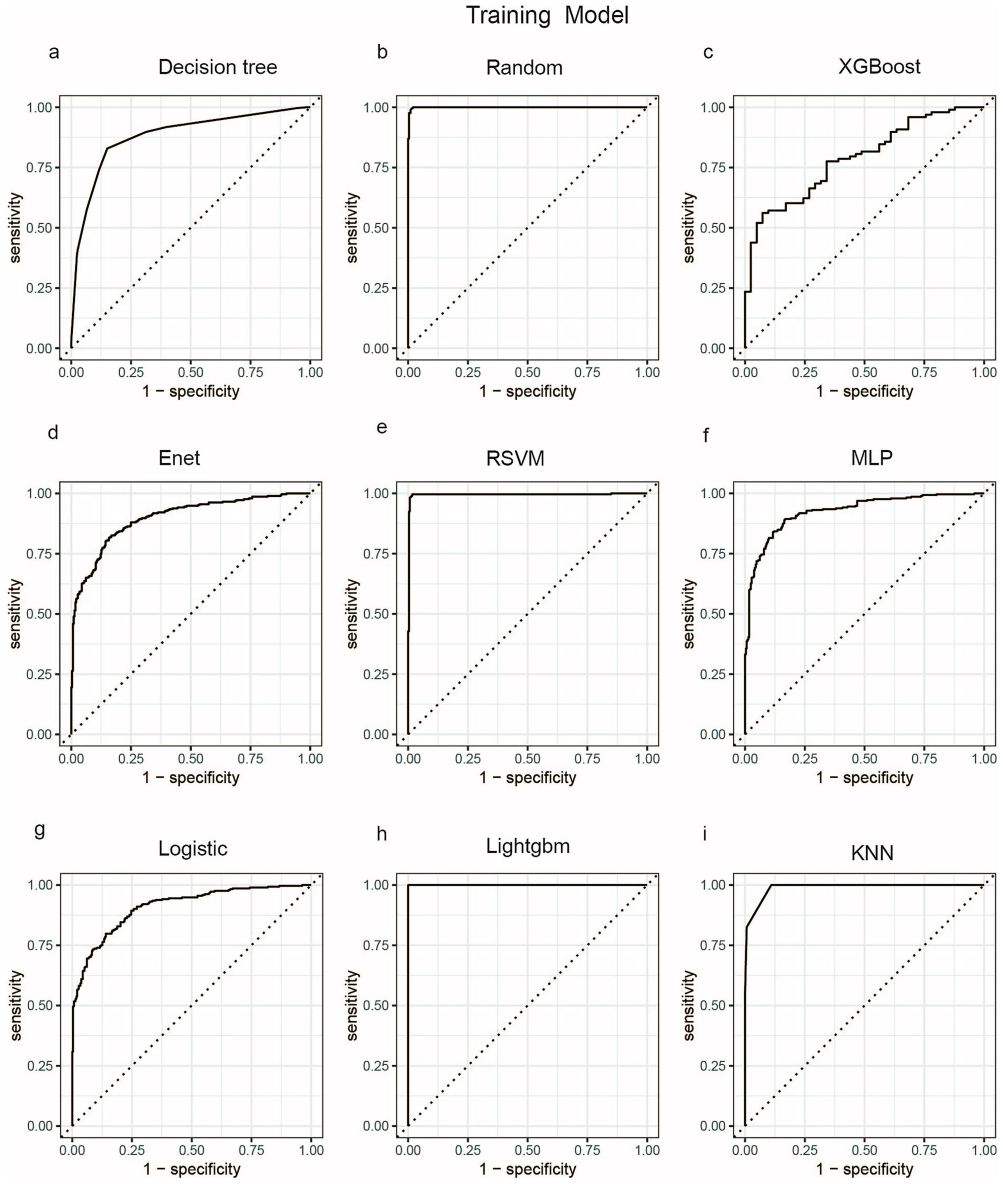
ROC curve analysis of predicting one-year mortality rate of patients with severe inflammatory bowel disease using machine learning algorithms in the train set. **(a)** Decision tree; **(b)** Random; **(c)** XGBoost; **(d)** Enet; **(e)** RSVM; **(f)** MLP; **(g)** Logistic; **(h)** LightGBM; **(i)** KNN. Random, Random Forest; XGBoost, Extreme Gradient Boosting; Enet, Elastic Net; RSVM, Reduced Support Vector Machine; MLP, Multilayer Perceptron; Logistic, logistic regression; LightGBM, Light Gradient Boosting Machine; KNN, K-Nearest Neighbor.

**Figure 4:**
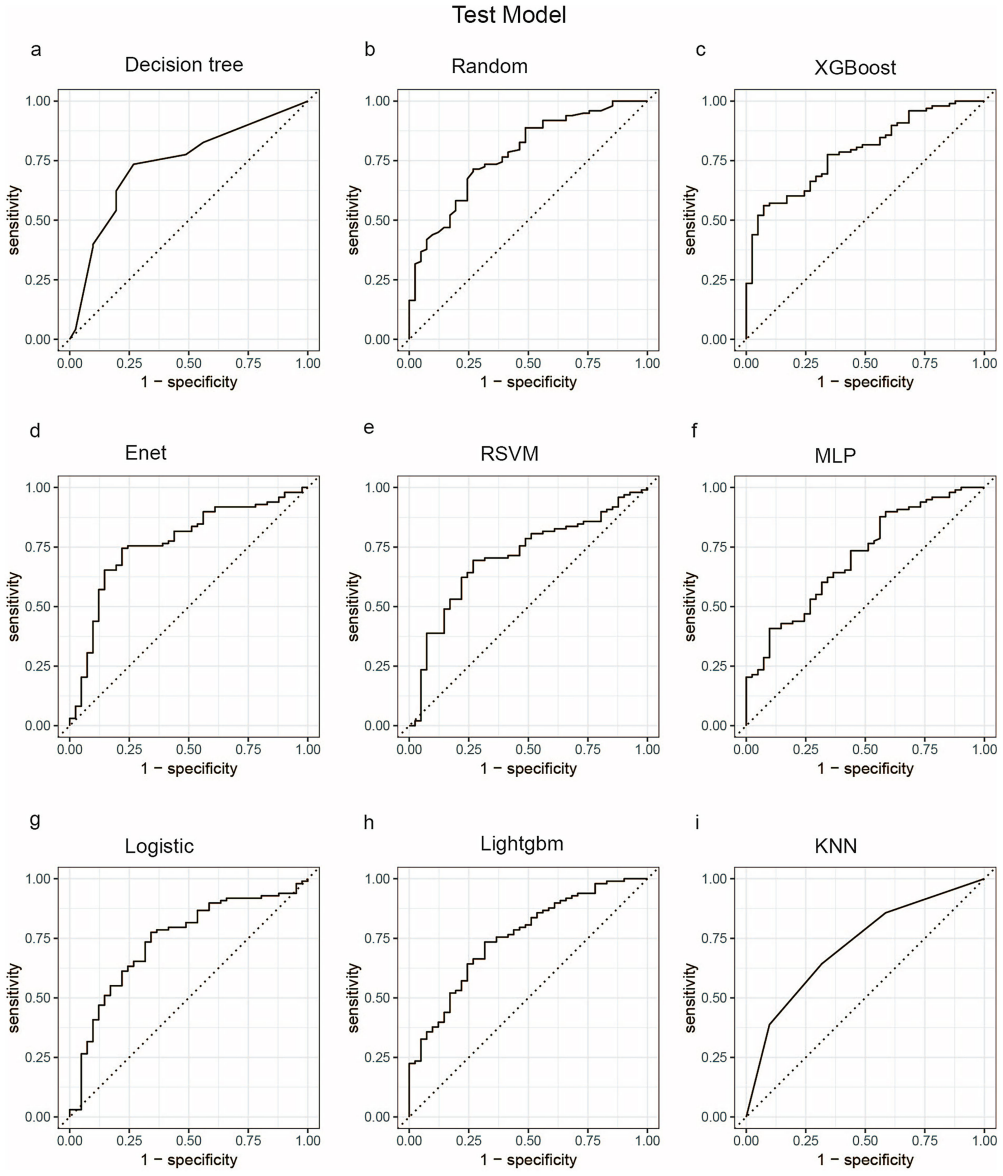
ROC curve analysis of predicting one-year mortality rate of patients with severe inflammatory bowel disease using machine learning algorithms in the test set. **(a)** Decision tree; **(b)** Random; **(c)** XGBoost; **(d)** Enet; **(e)** RSVM; **(f)** MLP; **(g)** Logistic; **(h)** LightGBM; **(i)** KNN. Random, Random Forest;XGBoost, Extreme Gradient Boosting; Enet, Elastic Net; RSVM, Reduced Support Vector Machine; MLP, Multilayer Perceptron; Logistic, logistic regression; LightGBM, Light Gradient Boosting Machine; KNN, K-Nearest Neighbor.

We employed a diverse range of ML algorithms to predict the long-term survival rate of critically ill IBD patients, including XGBoost, LightGBM, RF, DT, Enet, MLP, KNN, Logistic regression and RSVM. These ML algorithms were compared against a standard IBD prediction method. Initially, we utilized conventional stepwise logistic regression to assess predictive outcomes. For the ML models, we conducted repeated 5-fold cross-validation, optimizing the Area Under the Curve (AUC) to identify the best model parameters (22). The performance of the models was evaluated based on several metrics: AUC, sensitivity, specificity, positive predictive value (PPV), negative predictive value (NPV), and accuracy. Figures 3, 4 display the receiver operating characteristic (ROC) curves, which are used to assess each model’s predictive performance regarding long-term survival outcomes in critically ill IBD patients.

**Figure 5:**
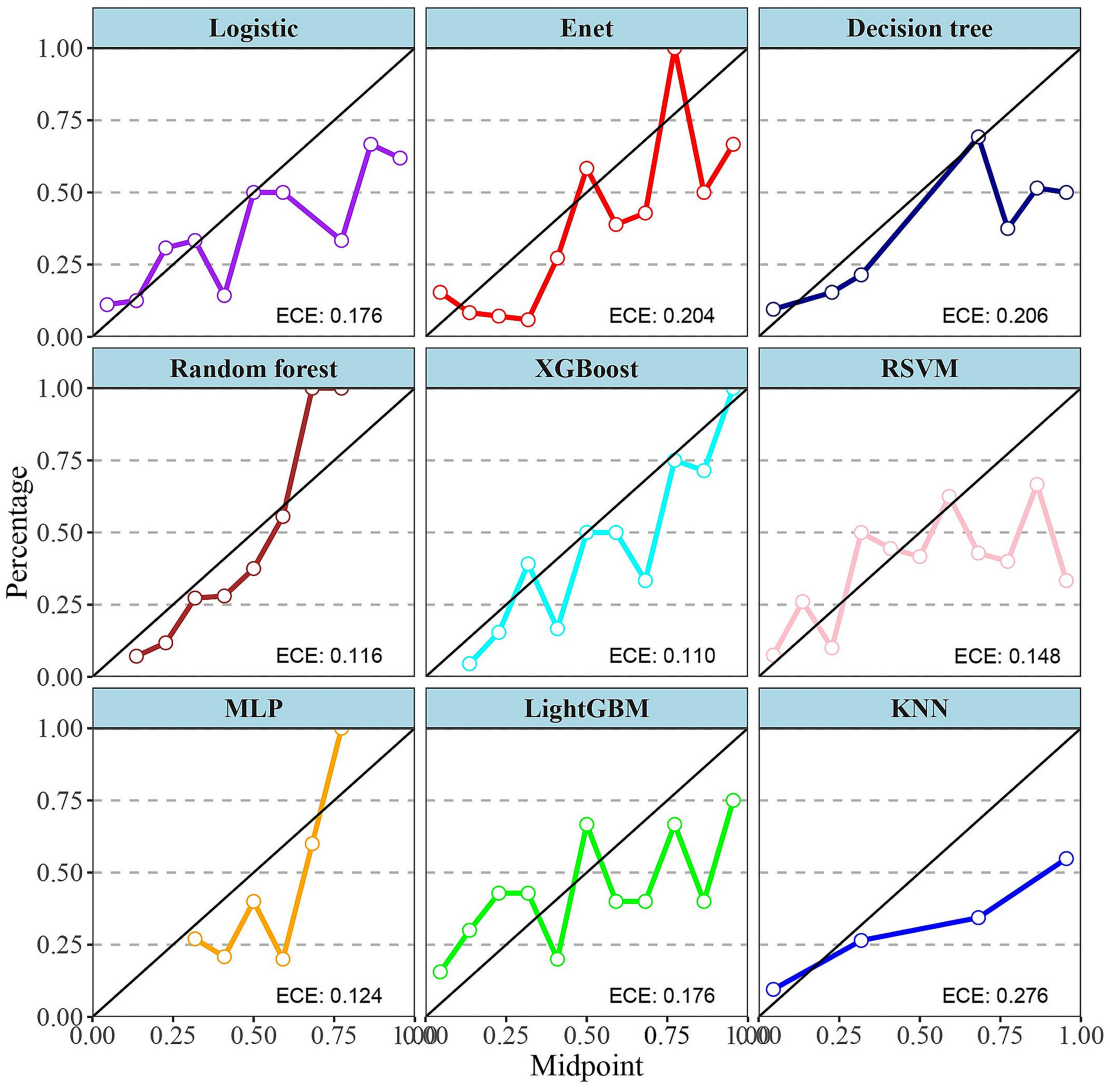
Calibration curves for nine different machine learning models. Calibration curves are used to assess the accuracy of the models’ predicted probabilities. Each subplot represents a model, with the *x*-axis being the midpoint of the predicted probabilities (Midpoint) and the *y*-axis being the percentage of actual outcomes (Percentage). XGBoost, Extreme Gradient Boosting; Enet, Elastic Net; RSVM, Reduced Support Vector Machine; MLP, Multilayer Perceptron; Logistic, logistic regression; LightGBM, Light Gradient Boosting Machine; KNN, K-Nearest Neighbor; ECE, expected calibration error.

The choice of the Random Forest (RF) model for identifying severe IBD patients rests primarily on its exceptional sensitivity in both the training and testing cohorts. Sensitivity is the key metric for minimizing missed diagnoses of severe cases. As shown in Table 2, RF achieved a perfect sensitivity of 1.00 in the training cohort, and among all algorithms it retained the highest sensitivity of 0.88 in the testing cohort, outperforming alternatives such as XGBoost (0.77) and logistic regression (0.74). This high sensitivity ensures reliable identification of severe cases, which is critical for timely clinical intervention. Notably, RF also exhibited excellent overall predictive performance, with an AUC of 0.78 and an accuracy of 0.76 in the testing cohort, indicating strong generalizability. Its Expected Calibration Error (ECE) of 0.116 (Figure 5) further underscores the reliability of its probability estimates, surpassing models such as KNN (ECE = 0.276) and Enet (ECE = 0.204). Compared with models like LightGBM, which displayed severe overfitting, RF effectively balanced fit and generalization. Moreover, RF displayed the shortest five-fold cross-validation error bars (Supplementary Figure 1), rendering it a robust choice for clinical applications where both sensitivity and stability are paramount.

**Table 2:** Predictive performance comparison of the nine types of machine learning algorithms.

Variables	Train Cohort					
	AUC	Accuracy	Sensitivity	Specificity	PPV	NPV
Logistic	0.91	0.83	0.80	0.86	0.85	0.81
Enet	0.90	0.83	0.82	0.85	0.84	0.82
DT	0.88	0.84	0.83	0.85	0.85	0.83
RF	1.00	0.99	1.00	0.98	0.98	1.00
XGBoost	0.98	0.94	0.92	0.95	0.95	0.93
RSVM	0.99	0.99	1.00	0.98	0.98	1.00
MLP	0.93	0.86	0.89	0.83	0.84	0.89
LightGBM	1.00	1.00	1.00	1.00	1.00	1.00
KNN	0.99	0.95	1.00	0.89	0.90	1.00

	Test cohort					
Logistic	0.74	0.72	0.74	0.66	0.84	0.52
Enet	0.77	0.74	0.74	0.73	0.87	0.55
DT	0.74	0.73	0.73	0.73	0.87	0.54
RF	0.78	0.76	0.88	0.49	0.80	0.63
XGBoost	0.79	0.73	0.77	0.66	0.84	0.54
RSVM	0.71	0.68	0.83	0.34	0.75	0.45
MLP	0.71	0.68	0.78	0.46	0.78	0.46
LightGBM	0.75	0.50	0.33	0.93	0.91	0.37
KNN	0.72	0.73	0.86	0.41	0.78	0.55

XGBoost, Extreme Gradient Boosting; Enet, Elastic Net; DT, Decision Tree; RSVM, Reduced Support Vector Machine; MLP, Multilayer Perceptron; Logistic, logistic regression; LightGBM, Light Gradient Boosting Machine; KNN, K-Nearest Neighbor.

**Figure 6:**
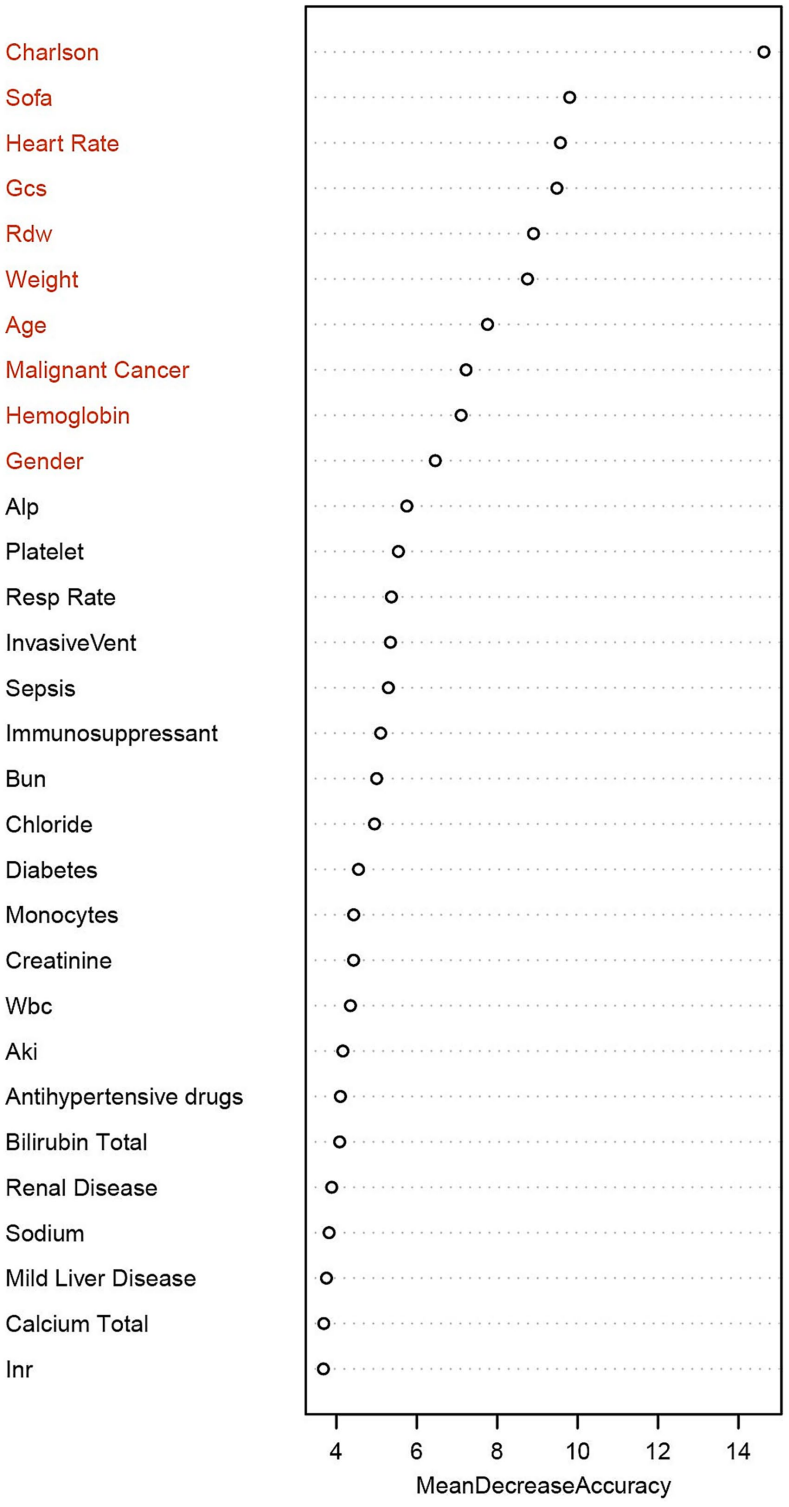
Ranking of the importance of clinical features based on the random forest model. Using “Mean Decrease Accuracy” as the measurement index, the length of the bar represents the average decrease of each variable in the prediction accuracy of the model. The top 10 key features are in sequence: Charlson Comorbidity Index, SOFA score, heart rate, GCS, Rdw, weight, age, malignant tumor, hemoglobin, and gender. Charlson, Charlson Comorbidity Index; Sofa, Sequential Organ Failure Assessment; Gcs, Glasgow Coma Scale; Rdw, Red Cell Distribution Width; Alp, Alkaline Phosphatase; Resp rate, Respiratory rate; InvasiveVent, Invasive Ventilation; Bun, Blood Urea Nitrogen; Wbc, White Blood Cell Count; Aki, Acute Kidney Injury; INR, International Normalized Ratio.

Finally, we selected the top 10 variables screened by the RF machine learning model as the final modeling indicators. As shown in Figure 6, these 10 variables are: CCI, Rdw, Gcs, Sofa, Age, Heart Rate, Weight, Malignant Cancer, Hemoglobin, and Gender.

### Screening of independent influencing factors based on multivariate logistic regression analysis

To identify the factors with independent prognostic impact among 10 predictors in the Random Forest (RF) machine learning model, we employed multivariable logistic regression analysis, as illustrated in Table 3. A total of nine factors were identified as having independent effects on prognosis. Specifically, for each 1-point increase in the CCI, the risk of the outcome increased by approximately 15% (odds ratio [OR], 1.15; 95% confidence interval [CI], 1.02–1.28). Similarly, the risk of the outcome increased with each unit increase in Rdw (OR, 1.11; 95% CI, 1.03–1.20), SOFA (OR, 1.10; 95% CI, 1.03–1.18), age (OR, 1.04; 95% CI, 1.01–1.06), and heart rate (OR, 1.02; 95% CI, 1.01–1.03). In contrast, an increase in GCS by 1 point was associated with a decreased risk of the outcome (OR, 0.92; 95% CI, 0.86–0.99), as was an increase in weight (OR, 0.98; 95% CI, 0.97–0.99) with each unit. Notably, patients with malignant cancer had a significantly higher risk of the target outcome, approximately 2.42 times that of patients without malignant cancer (OR, 2.42; 95% CI, 1.18–4.96). Additionally, compared with female patients, male patients had a significantly lower risk, with a 39% reduction (OR, 0.61; 95% CI, 0.39–0.96).

**Table 3:** Univariate and multivariate logistic regression analysis.

Variables	Univariate		Multivariate	
	*P*	OR (95%CI)	*P*	OR (95%CI)
Charlson	<0.001	1.30 (1.21 ~ 1.40)	0.018	1.15 (1.02 ~ 1.28)
Rdw	<0.001	1.15 (1.08 ~ 1.22)	0.007	1.11 (1.03 ~ 1.20)
Gcs	<0.001	0.88 (0.83 ~ 0.92)	0.024	0.92 (0.86 ~ 0.99)
Sofa	<0.001	1.14 (1.09 ~ 1.19)	0.003	1.10 (1.03 ~ 1.18)
Age	<0.001	1.04 (1.03 ~ 1.05)	0.002	1.04 (1.01 ~ 1.06)
Heart rate	<0.001	1.02 (1.01 ~ 1.03)	<0.001	1.02 (1.01 ~ 1.03)
Weight	<0.001	0.98 (0.97 ~ 0.99)	0.001	0.98 (0.97 ~ 0.99)
Malignant cancer				
No		1.00 (Reference)		1.00 (Reference)
Yes	<0.001	4.27 (2.42 ~ 7.53)	0.016	2.42 (1.18 ~ 4.96)
Gender				
Female		1.00 (Reference)		1.00 (Reference)
Male	0.004	0.58 (0.40 ~ 0.84)	0.034	0.61 (0.39 ~ 0.96)
Hemoglobin	0.003	0.88 (0.81 ~ 0.96)		

Charlson, Charlson Comorbidity Index; Rdw, Red Cell Distribution Width; Sofa, Sequential Organ Failure Assessment; Gcs, Glasgow Coma Scale.

### SHAP analysis of key determinants of mortality risk

This study combines the SHAP framework with predictive analysis to systematically evaluate the key determinants of mortality risk through quantitative feature importance analysis. Previously identified important clinical predictors include CCI, Rdw, Gcs, Sofa, age, heart rate, weight, malignant cancer, and gender. In addition, detailed trend analysis reveals the correlation between biomarkers and mortality risk.

**Figure 7:**
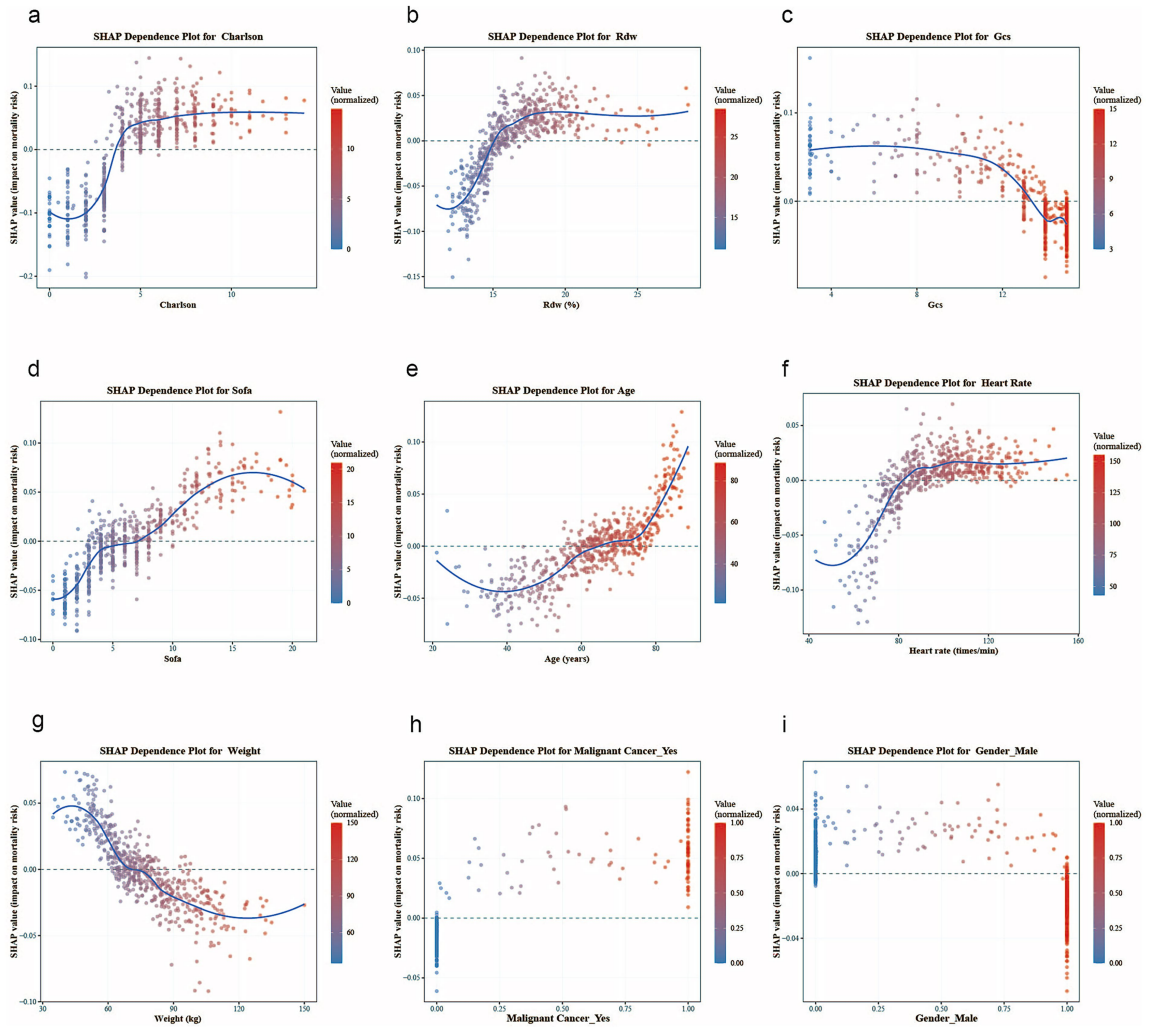
SHAP dependence plots for key variables, with each plot corresponding to the following: **(a)** Charlson Comorbidity Index, **(b)** Rdw, **(c)** GCS, **(d)** sofa, **(e)** age, **(f)** heart rate, **(g)** weigh, **(h)** malignant tumor, and **(i)** gender.

Figure 7 further illustrates specific patterns: the Charlson index, Rdw, and SOFA score show a significant monotonic positive correlation with mortality risk. The SHAP value for heart rate remains stable within the normal range (60–100 beats per minute), but the risk contribution increases significantly outside this range (<60 or >100 beats per minute). The SHAP value associated with body weight is close to neutral in the moderate range (60–100 kg), while both low body weight (<60 kg) and high body weight (>100 kg) are associated with increased risk. For age, the SHAP value rises exponentially after ≥65 years old. A GCS score <9 corresponds to a sharp deterioration in the SHAP value, indicating a significant increase in mortality risk in patients with severe neurological impairment. Furthermore, being male is associated with a reduced mortality risk, while concurrent malignant tumor is the most critical risk factor.

This explainable machine learning approach not only quantifies the relative importance of predictors but also visually demonstrates how different value ranges of each biomarker uniquely influence mortality risk (23), providing actionable insights for clinicians. The framework enables precise risk stratification based on patients’ specific laboratory parameters and vital signs, facilitates targeted interventions for abnormal parameter ranges, and supports dynamic monitoring of treatment responses.

### Development and validation of models

**Figure 8:**
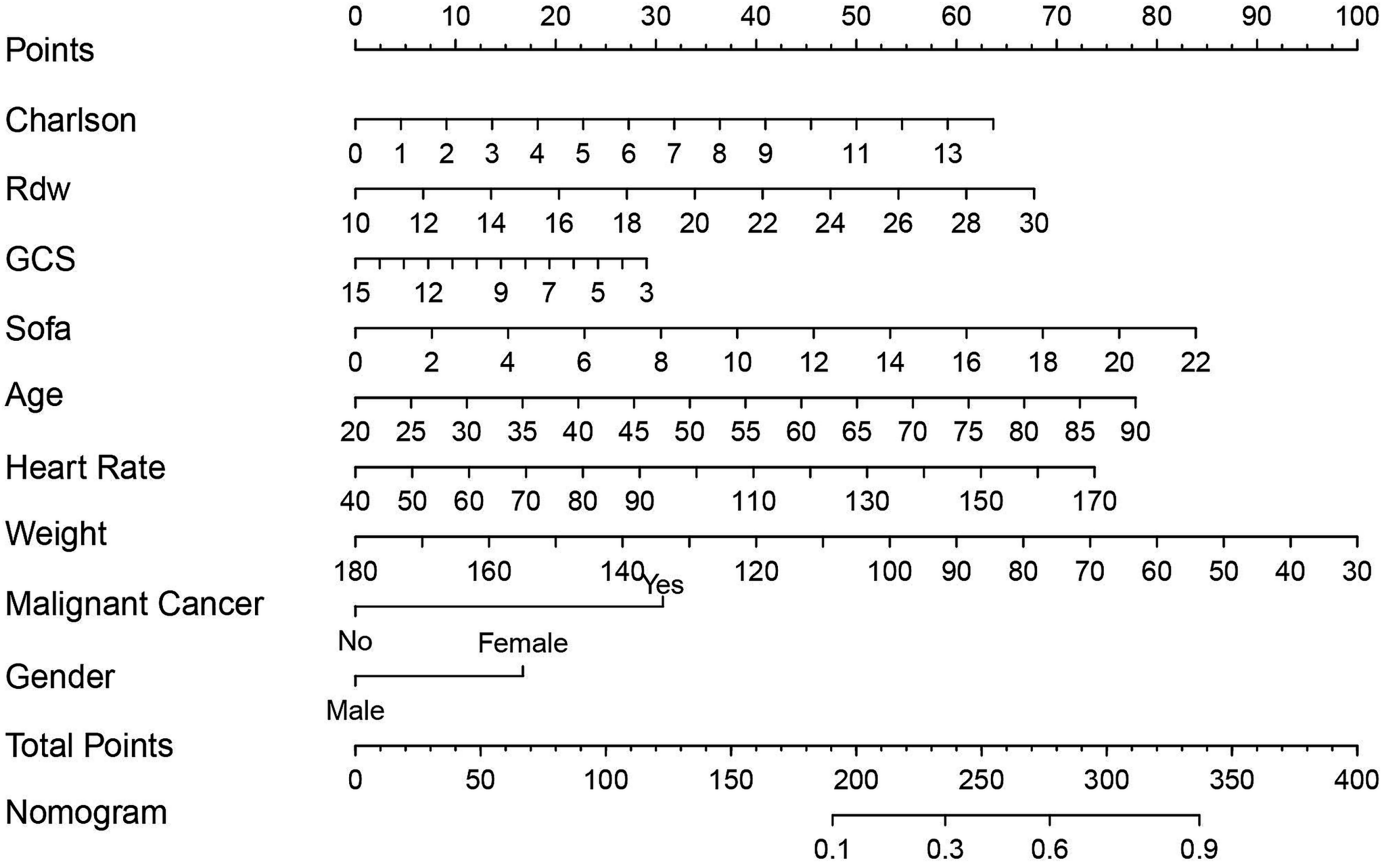
The Nomogram used for predicting the one-year mortality rate of severe inflammatory bowel disease is established using the Logistic regression algorithm. The final score (i.e., total points) calculated as the sum of individual scores of the 7 variables included in the Nomogram. Sofa, Sequential Organ Failure Assessment; Charlson, Charlson Comorbidity Index; Gcs, Glasgow Coma Scale; Rdw, Red Cell Distribution Width.

Based on stepwise regression and SHAP analyses, we identified nine pivotal variables—CCI, Rdw, GCS, SOFA, age, heart rate, weight, malignant cancer, and gender—to construct the nomogram (Figure 8). Compared with traditional logistic regression equations, this nomogram offers superior clinical practicality. By assigning scores to each risk factor, summing the total points, and mapping the total to the adverse-outcome risk axis, clinicians can reliably estimate the 1-year mortality risk in patients with inflammatory bowel disease (IBD); higher total scores indicate worse prognosis.

**Figure 9:**
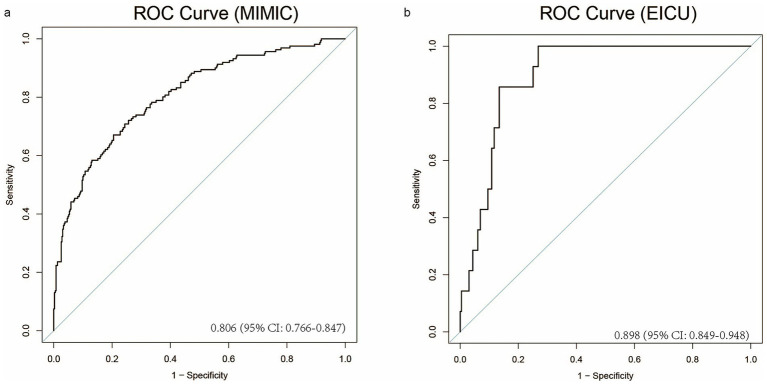
The model’s performance is evaluated on the MIMIC and EICU datasets through ROC curves. **(a)** The ROC curve for the MIMIC dataset, **(b)** The ROC curve for the EICU dataset.

To assess model performance, we calculated the C-index. As shown in Figure 9A, the C-index reached 0.806 (95% CI: 0.766–0.847), demonstrating robust stability and reliability. Furthermore, external validation was performed using an independent cohort of critically ill IBD patients from the eICU database. Figure 9B shows a C-index of 0.898 (95% CI: 0.849–0.948), confirming the model’s high predictive accuracy.

## Discussion

In the prognostic research of IBD, the Mayo Score, Ulcerative Colitis Endoscopic Severity Index (UCEIS), Crohn’s Disease Endoscopic Severity Score (SES-CD), as well as the assessment of mucosal inflammation by colonoscopy, have been widely recognized as the “gold standard” and effective prognostic indicators for judging the disease activity of IBD patients (24). However, colonoscopy is not only costly but also poses a relatively high risk for critically ill patients, which severely limits its application in critically ill IBD patients in the ICU (25). Currently, studies on the prognostic factors related to critically ill IBD patients remain relatively scarce. According to the data from the Intensive Care Unit of the University Medical Center Hamburg - Eppendorf, among all the IBD patients admitted between 2013 and 2022, 16.3% of them died within 90 days of admission (10). In the Intensive Care Unit of Mount Sinai Medical Center, among the 95 patients with ulcerative colitis or Crohn’s disease admitted between 2003 and 2008, the overall 30-day mortality rate was as high as 18.9%, and the increase in mortality was closely associated with factors such as mechanical ventilation, vasoactive drug requirements, severe sepsis, acute kidney injury, APACHE II score, hypoalbuminemia, and thromboembolism (26). At present, accurately identifying the long-term prognosis of critically ill IBD patients still faces numerous difficulties.

This study, through a retrospective cohort study combined with multiple machine learning techniques, constructed a model for predicting the long-term survival rate of critically ill IBD patients based on the two major databases, MIMIC and eICU. This model can accurately identify patients with a high risk of death within 1 year, enabling clinicians to take intervention measures such as optimizing treatment regimens and strengthening monitoring in advance. It is expected to significantly reduce the 1-year mortality rate of patients and improve their long-term prognosis. In addition, the individualized risk assessment function of the model can provide a precise basis for stratified management for the medical team, thereby enhancing the overall medical efficiency and quality. By further validating this model on external data sets, we can enhance its universality among different populations and clinical settings, further highlighting its important clinical value in the management of IBD patients in the ICU, and providing strong support for optimizing treatment strategies and improving patient prognosis.

A total of 551 patients were included in this study. Through the construction and validation of the model, we determined the main predictive factors for the prognosis of IBD patients in the ICU, including the CCI, Rdw, Gcs, Sofa, age, heart rate, weight, malignant cancer and gender. Patients with a long history of IBD may have an increased cancer risk due to the chronic inflammation of the intestinal mucosa, the extraintestinal manifestations of the disease, and the immunosuppressive treatment for IBD (27). Besides colorectal cancer (28), this increased risk is also related to other malignancies, such as hematological malignancies (29) and carcinoid tumors (30). In clinical settings, when dealing with IBD patients who have active cancer, the determination of whether to initiate or sustain the use of biologics ought to be made through a multidisciplinary methodology (31). Malignant tumors may exacerbate adverse outcomes in patients (31, 32). The SOFA score dynamically reflects the progression of MODS by quantifying the degree of dysfunction in six major systems: respiratory, coagulation, hepatic, circulatory, neurological, and renal (33). In critically ill IBD patients, this score can effectively identify organ function deterioration caused by complications such as septic shock and toxic megacolon.

There is an important correlation between heart rate variability and fatigue in patients with IBD (34). Specifically, a decreased heart rate variability may make patients more prone to fatigue, which can be caused by factors such as nutritional deficiencies, inflammation, and poor sleep (34). And the heart rate recovery of patients with IBD occurs during the clinical remission period (35). There is a definite connection between IBD and malnutrition. Protein-energy malnutrition and micronutrient deficiencies may promote inflammation through malnutrition (36). Although body weight is not the sole indicator for assessing malnutrition, in the intensive care unit, it can, to some extent, reflect the nutritional status of patients (37, 38).

A systematic review demonstrated that approximately 25–30% of the IBD population are aged 60 years or older (39). Among inpatients, elderly individuals are at a higher risk of adverse outcomes (40), and advanced age (≥65 years) is associated with increased in-hospital mortality in IBD (41). Among 218 IBD patients with an equal gender distribution, female IBD patients were significantly more susceptible to IBD-related symptoms than male patients (42). Data from the Global Burden of Disease Study (GBD) revealed that from 1990 to 2019, female patients with IBD had higher mortality rates both in the United States and globally (43). The highest age-standardized death rate (ASDR) was observed in women in the United States, at 1.08 (APC: 1.28; 95% CI 1.19 to 1.38) (43).

As early as the 4th Congress of the European Crohn’s and Colitis Organisation, it was proposed that RDW is a new diagnostic and activity marker for inflammatory bowel disease (44). A multicenter meta-analysis showed that in Crohn’s disease patients, RDW levels were significantly higher in active cases than in inactive ones (*p* = 0.007), and the same trend was observed in ulcerative colitis (*p* = 0.02) (45). Moreover, the association between increased RDW and active IBD is evident in both IBD patients with and without anemia (46). The GCS score is frequently used in the intensive care unit to objectively assess patients’ level of consciousness (47). A higher total score on the GCS indicates a better state of consciousness for the patient (48), including severe IBD. However, in patients with post-traumatic IBD, a higher admission GCS score may expose them to greater risks of surgery and complications after trauma.

A progressive increase in CCI is correlated with a stepwise rise in mortality. The CCI is also characterized by the clinimetric property of incremental validity, meaning that adding CCI to other measurements can enhance the overall predictive accuracy (49). It has been demonstrated to predict long-term mortality in different clinical populations, including those in internal medicine, surgery, and the intensive care unit (ICU) (50). By harnessing the advancements in computer science and clinical informatics and integrating them with dynamic clinical parameters, it is likely to yield a more favorable clinical risk prediction model compared to merely depending on a single static clinical parameter. The prediction model developed in this study formulates a visual nomogram, enabling an intuitive forecast of the risk of Severe IBD.

Our research has several limitations. First, it is a retrospective investigation, which inevitably introduces retrospective bias. Therefore, future studies should adopt a more rigorous prospective design. Second, anti-TNF therapy is critically important for managing IBD (51). Nevertheless, as it is not distinctly identified in MIMIC IV, additional studies are essential to determine its potential impact on our findings. Third, while our newly developed model showed encouraging predictive accuracy in both internal and external validation cohorts of MIMIC IV and EICU, concerns about its scalability to other healthcare facilities remain, given that the external validation cohort’s performance was not as strong as that of the internal cohort. Therefore, a larger external validation sample is crucial for substantiating the efficacy of our model. Despite these drawbacks, our study indicates that the model we developed holds significant potential and deserves further investigation in subsequent clinical practices and research endeavors.

## Conclusion

We’ve developed a predictive model based on 9 key variables. It can fairly accurately evaluate the likelihood of long-term mortality in severe IBD patients, helping to early identify those at high risk of death upon admission. The model provides clinicians with an actionable tool for early risk stratification, enabling targeted interventions to improve outcomes. The combination of ML algorithms and traditional statistical methods enhances translational potential, addressing a critical gap in IBD critical care prognostication.

## Data Availability

The original contributions presented in the study are included in the article/Supplementary material, further inquiries can be directed to the corresponding author/s.

## Supplementary material

SUPPLEMENTARY FIGURE 1 Comparison of cross-validated AUCs (areas under the curve) among nine machinelearning models for predicting ICU mortality in patients with IBD. Data points represent the mean values from 5-fold cross-validation, and error bars indicate the standard error (mean ± SE).
