# Supplementary figures and images for: Random forest-driven mortality prediction in critical IBD care: a dual-database model integrating comorbidity patterns and real-time physiometrics
Source: Front Med (Lausanne). 2025 Aug 8;12:1624899. doi: 10.3389/fmed.2025.1624899 (PMC12370684; doi:10.3389/fmed.2025.1624899)

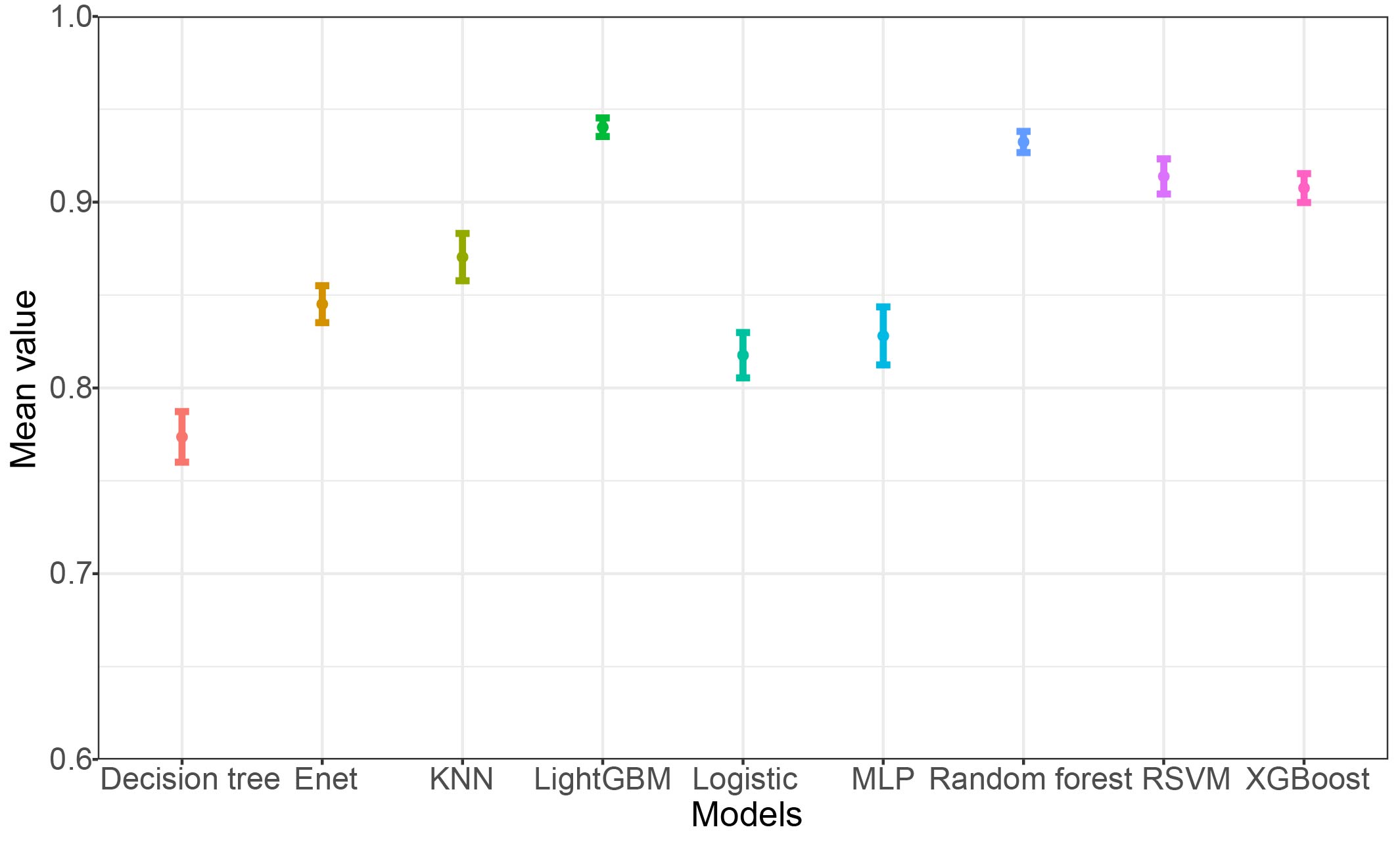

Supplement: SUPPLEMENTARY FIGURE 1 — Comparison of cross-validated AUCs (areas under the curve) among nine machinelearning models for predicting ICU mortality in patients with IBD. Data points represent the mean values from 5-fold cross-validation, and error bars indicate the standard error (mean ± SE). [file Image_1.jpeg]
