# Supplementary material for: Random forest-driven mortality prediction in critical IBD care: a dual-database model integrating comorbidity patterns and real-time physiometrics
Source: Front Med (Lausanne). 2025 Aug 8;12:1624899. doi: 10.3389/fmed.2025.1624899 (PMC12370684; doi:10.3389/fmed.2025.1624899)
Supplement: Supplementary file 2 [file Table_1.docx]

Supplementary table 1 Baseline Characteristics of Training and Testing Groups

|  | Test  （N=139） | Train  （N=412） | p.overall |  |
| --- | --- | --- | --- | --- |
| **Demographic** |  |  |  |  |
| Mortality365 (%): |  |  | 1 |  |
| No | 98 (70.5%) | 292 (70.9%) |  |  |
| Yes | 41 (29.5%) | 120 (29.1%) |  |  |
| Age (years) | 63.4 (15.3) | 61.9 (14.4) | 0.331 |  |
| Gender (%): |  |  | 0.309 |  |
| Female | 70 (50.4%) | 185 (44.9%) |  |  |
| Male | 69 (49.6%) | 227 (55.1%) |  |  |
| Smoker (%): |  |  | 0.665 |  |
| No | 127 (91.4%) | 383 (93.0%) |  |  |
| Yes | 12 (8.63%) | 29 (7.04%) |  |  |
| Alcohol abuse(%): |  |  | 0.265 |  |
| No | 137 (98.6%) | 410 (99.5%) |  |  |
| Yes | 2 (1.44%) | 2 (0.49%) |  |  |
| **Comorbidities** |  |  |  |  |
| Hypertension (%): |  |  | 1 |  |
| No | 95 (68.3%) | 283 (68.7%) |  |  |
| Yes | 44 (31.7%) | 129 (31.3%) |  |  |
| Diabetes (%): |  |  | 0.232 |  |
| No | 100 (71.9%) | 319 (77.4%) |  |  |
| Yes | 39 (28.1%) | 93 (22.6%) |  |  |
| Mild liver disease (%): |  |  | 0.608 |  |
| No | 104 (74.8%) | 319 (77.4%) |  |  |
| Yes | 35 (25.2%) | 93 (22.6%) |  |  |
| Severe liver disease (%): |  |  | 0.508 |  |
| No | 117 (84.2%) | 358 (86.9%) |  |  |
| Yes | 22 (15.8%) | 54 (13.1%) |  |  |
| Renal disease (%): |  |  | 1 |  |
| No | 110 (79.1%) | 327 (79.4%) |  |  |
| Yes | 29 (20.9%) | 85 (20.6%) |  |  |
| Malignant cancer (%): |  |  | 0.777 |  |
| No | 126 (90.6%) | 368 (89.3%) |  |  |
| Yes | 13 (9.35%) | 44 (10.7%) |  |  |
| Rheumatic disease (%): |  |  | 0.81 |  |
| No | 132 (95.0%) | 387 (93.9%) |  |  |
| Yes | 7 (5.04%) | 25 (6.07%) |  |  |
| Sepsis (%): |  |  | 0.92 |  |
| No | 55 (39.6%) | 167 (40.5%) |  |  |
| Yes | 84 (60.4%) | 245 (59.5%) |  |  |
| Clostridium difficile infection (%): | |  |  | 0.13 |
| No | | 120 (86.3%) | 376 (91.3%) |  |
| Yes | | 19 (13.7%) | 36 (8.74%) |  |
| Black stool (%): | |  |  | 0.624 |
| No | | 134 (96.4%) | 391 (94.9%) |  |
| Yes | | 5 (3.60%) | 21 (5.10%) |  |
| Intestinal dysfunction (%): | |  |  | 1 |
| No | | 138 (99.3%) | 407 (98.8%) |  |
| Yes | | 1 (0.72%) | 5 (1.21%) |  |
| Aki (%): |  |  | 0.851 |  |
| No | 41 (29.5%) | 127 (30.8%) |  |  |
| Yes | 98 (70.5%) | 285 (69.2%) |  |  |
| **Treatments** |  |  |  |  |
| InvasiveVent (%): |  |  | 0.087 |  |
| No | 95 (68.3%) | 246 (59.7%) |  |  |
| Yes | 44 (31.7%) | 166 (40.3%) |  |  |
| Cpr (%): |  |  | 1 |  |
| No | 138 (99.3%) | 410 (99.5%) |  |  |
| Yes | 1 (0.72%) | 2 (0.49%) |  |  |
| Rrt (%): |  |  | 1 |  |
| No | 124 (89.2%) | 368 (89.3%) |  |  |
| Yes | 15 (10.8%) | 44 (10.7%) |  |  |
| Immunosuppressant (%): |  |  | 0.772 |  |
| No | 124 (89.2%) | 373 (90.5%) |  |  |
| Yes | 15 (10.8%) | 39 (9.47%) |  |  |
| Corticosteroids (%): |  |  | 0.879 |  |
| No | 83 (59.7%) | 241 (58.5%) |  |  |
| Yes | 56 (40.3%) | 171 (41.5%) |  |  |
| Antihypertensive drugs (%): |  |  | 0.268 |  |
| No | 46 (33.1%) | 160 (38.8%) |  |  |
| Yes | 93 (66.9%) | 252 (61.2%) |  |  |
| Colonoscopy (%): |  |  | 1 |  |
| No | 134 (96.4%) | 396 (96.1%) |  |  |
| Yes | 5 (3.60%) | 16 (3.88%) |  |  |
| **Vital signs and Laboratory results** |  |  |  |  |
| Temperature (℃) | 36.7 (0.70) | 36.7 (0.97) | 0.909 |  |
| Heart rate (times/min) | 92.8 (21.8) | 93.7 (21.5) | 0.675 |  |
| Resp rate (times/min) | 19.5 (5.98) | 20.2 (6.19) | 0.203 |  |
| Sbp (mmHg) | 117 (22.5) | 117 (21.6) | 0.954 |  |
| Dbp (mmHg) | 66.6 (17.6) | 67.2 (15.6) | 0.726 |  |
| Wbc (×10^9^/L) | 13.6 (9.82) | 12.8 (13.4) | 0.459 |  |
| Eosinophils abs (K/uL) | 0.11 (0.17) | 0.08 (0.15) | 0.098 |  |
| Basophils abs (K/uL) | 0.03 (0.03) | 0.02 (0.05) | 0.942 |  |
| Lymphocytes abs (K/uL) | 1.16 (0.92) | 1.09 (1.23) | 0.494 |  |
| Neutrophils abs (K/uL) | 11.7 (8.96) | 10.5 (7.95) | 0.181 |  |
| Monocytes abs (K/uL) | 0.74 (0.79) | 0.67 (1.05) | 0.428 |  |
| Eosinophils (%) | 0.90 (1.46) | 0.89 (1.73) | 0.912 |  |
| Monocytes (%) | 5.87 (4.91) | 5.70 (5.32) | 0.726 |  |
| Neutrophils (%) | 76.9 (15.4) | 77.6 (16.5) | 0.656 |  |
| Basophils (%) | 0.23 (0.28) | 0.24 (0.42) | 0.705 |  |
| Lymphocytes (%) | 11.9 (12.0) | 11.7 (11.5) | 0.835 |  |
| Rdw (%) | 15.7 (2.85) | 16.1 (2.83) | 0.252 |  |
| Platelet (×10^9^/L) | 219 (139) | 227 (146) | 0.562 |  |
| Creatinine (mg/dL) | 1.51 (1.56) | 1.65 (2.01) | 0.401 |  |
| Bun (mg/dL) | 30.2 (29.2) | 28.5 (25.8) | 0.551 |  |
| Anion gap (mEq/L) | 15.0 (5.22) | 14.5 (4.84) | 0.279 |  |
| Calcium total (mg/dL) | 8.23 (0.96) | 8.20 (1.04) | 0.785 |  |
| Pt (sec) | 18.6 (10.6) | 17.8 (12.3) | 0.487 |  |
| inr | 1.70 (0.97) | 1.67 (1.20) | 0.777 |  |
| Alt (IU/L) | 125 (474) | 60.6 (127) | 0.116 |  |
| Ast (IU/L) | 222 (1037) | 101 (250) | 0.177 |  |
| Alp (IU/L) | 124 (118) | 128 (124) | 0.731 |  |
| Bilirubin total (mg/dL) | 2.43 (5.85) | 2.63 (5.73) | 0.717 |  |
| Chloride (mg/dL) | 103 (8.09) | 103 (7.28) | 0.617 |  |
| Glucose (mg/dL) | 150 (104) | 141 (71.3) | 0.304 |  |
| Potassium (mg/dL) | 4.15 (0.83) | 4.04 (0.74) | 0.16 |  |
| Sodium (mg/dL) | 137 (5.83) | 136 (5.60) | 0.243 |  |
| Hemoglobin (g/dL) | 10.5 (2.44) | 10.2 (2.26) | 0.109 |  |
| Magnesium (g/dL) | 2.03 (0.56) | 1.93 (0.45) | 0.059 |  |
| Phosphate (g/dL) | 3.78 (1.51) | 3.73 (1.79) | 0.733 |  |
| Weight (kg) | 80.5 (23.5) | 78.4 (19.5) | 0.349 |  |
| sofa | 5.83 (4.36) | 5.96 (4.30) | 0.771 |  |
| gcs | 12.8 (3.53) | 13.0 (3.31) | 0.422 |  |
| charlson | 4.78 (2.74) | 4.42 (2.82) | 0.182 |  |

Abbreviations: Aki, Acute Kidney Injury; InvasiveVent, Invasive Ventilation;Cpr, Cardiopulmonary Resuscitation ;Rrt, Renal Replacement Therapy; Sbp, Systolic Blood Pressure; Dbp, Diastolic Blood Pressure; Wbc, White Blood Cell Count; Platelet, Rdw,Red Cell Distribution Width; Bun, Blood Urea Nitrogen; Pt, Partial Thromboplastin Time; INR, International Normalized Ratio; Alt, Alanine Aminotransferase; Ast, Aspartate Aminotransferase; Alp, Alkaline Phosphatase; Sofa, Sequential Organ Failure Assessment;Charlson, Charlson Comorbidity Index.
